# MTSA-SC: A multi-task learning approach for individual trip destination prediction with multi-trajectory subsequence alignment and space-aware loss functions

**DOI:** 10.1371/journal.pone.0325471

**Published:** 2025-06-06

**Authors:** Dan Luo, Fang Zhao, Hao Zhou, Chenxing Wang, Hao Xiong

**Affiliations:** 1 The School of Information Science and Technology, School of Artificial Intelligence, Beijing Forestry University, Beijing, China; 2 The School of Computer Science (National Pilot Software Engineering School), Beijing University of Posts and Telecommunications, Beijing, China; National University of Defense Technology, CHINA

## Abstract

Individual Trip Destination Prediction aims to accurately forecast an individual’s future travel destinations by analyzing their historical trajectory data, holding significant application value in intelligent navigation, personalized recommendations, and urban traffic management. However, challenges such as data sparsity, low quality, and complex spatiotemporal volatility pose substantial difficulties for prediction tasks. Existing studies exhibit notable limitations in insufficient integration of sparsity handling and prediction tasks, constrained modeling capability for local volatility, and inadequate exploration of fine-grained spatial dependencies, struggling to balance global patterns and local features in trajectory data. To address these issues, this paper proposes an individual trip destination prediction method that integrates multi-task learning, a multi-trajectory subsequence alignment attention mechanism, and a spatially consistent constrained cross-entropy loss function. Leveraging a multi-task learning framework(MTSA-SC), our approach collaboratively addresses trajectory recovery and prediction tasks, enhancing prediction accuracy while improving robustness to missing data. The multi-trajectory subsequence alignment attention mechanism incorporates sliding windows and convolutional operations to dynamically capture local volatility and diverse patterns in trajectories. The spatially consistent constrained loss function strengthens spatial feature learning through differential error penalty adjustments. Experimental results on public datasets from Shenzhen and Xiamen demonstrate recall rates of 0.722 and 0.6 under complete and sparse trajectory scenarios, respectively, outperforming state-of-the-art baselines by an average of 15.64%. This research provides robust technical support for intelligent travel recommendations and traffic management.

## Introduction

The rapid advancement of spatial-temporal data analytics has revolutionized decision-making in both environmental and urban sectors [1,2], with sensing technologies providing unprecedented insights into the interactions between humans and their environment. As globalization and urbanization continue to accelerate, these technological innovations have become critical for addressing the complex challenges of intelligent transportation systems [3]. The growing demand for accurate travel behavior prediction now serves two primary objectives: enhancing user experience through personalized services and enabling urban systems to achieve key goals, such as emission reduction and energy efficiency. These challenges lie at the heart of contemporary spatial-temporal modeling research, driving the need for innovative methodologies to optimize both individual mobility [4] and sustainable urban development [5].

Despite the potential of spatial-temporal modeling technologies, the acquisition and utilization of individual travel trajectory data remain fraught with challenges. Privacy concerns [6], device energy consumption [7], and data collection costs all contribute to the difficulty in obtaining high-quality, complete travel trajectory data [8]. As a result, the available data is often sparse and of low quality, complicating efforts to fully capture the intricacies of user travel behavior. Moreover, the inherent complexities of trajectory data—such as irregularities, local fluctuations, and diverse trajectory segments—further complicate the task of accurate destination prediction. These challenges underscore the need for more refined and robust approaches in the research and development of individual travel destination prediction models.

A significant body of research has focused on travel behavior prediction [9], evolving through two primary paradigms: traditional machine learning methods and contemporary deep learning models [10]. Conventional machine learning approaches for next location prediction rely on user behavior and contextual data, utilizing models such as linear regression, decision trees, and k-nearest neighbors [11]. While these methods can capture basic patterns, they often fall short in modeling the complex, dynamic nature of real-world mobility. To address these limitations, deep learning techniques, including Recurrent Neural Networks (RNNs) [12], Deep Convolutional Neural Networks (CNN) [13], Graph Neural Networks (GNNs) [14], and Transformer-based architectures [15], have been introduced. These methods offer improved performance by better capturing spatiotemporal dependencies, making them more suitable for handling the intricacies of real-world travel behavior.

However, both methodological strands exhibit fundamental limitations that hinder their practical application. Traditional models struggle with representing heterogeneous mobility patterns across different demographic groups, capturing long-range temporal dependencies in urban mobility systems, and accurately modeling spatial connectivity. While Deep learning methods partially address some of these issues through hierarchical feature extraction, they face significant challenges when confronted with real-world data imperfections such as data sparsity, missing sensor records, and heterogeneous noise. This dual limitation manifests through critical architectural flaws: TrajBERT’s [16] artificial continuity generation via uniform sampling distorts behavioral semantics while propagating measurement errors, DeepMove’s [17] attention mechanisms disintegrate under fragmented inputs by fixating on residual artifacts, and hybrid frameworks like MTrajRec [18] misclassify legitimate detours through rigid spatial constraints.Even meta-learning adaptations such as MetaTraj [19] exhibit paradoxical degradation—their adaptive updates amplify rather than suppress data flaws under extreme sparsity.

The interplay between methodological limitations creates self-reinforcing deterioration patterns: synthetic continuity mechanisms mask authentic sparsity characteristics, fragmented temporal contexts amplify residual noise artifacts, and rigid spatial constraints erroneously suppress legitimate behavioral deviations. This culminates in an irreconcilable modeling paradox - urban mobility prediction requires simultaneous preservation of spontaneous movement patterns from sparse observations and discriminative suppression of entangled sensor noise, objectives that existing architectures fundamentally conflict in dynamic environments.

These compounded limitations manifest through three core challenges for individual travel prediction:

1. **Asynchrony between mobility rhythm and pattern variability:** Individual travel trajectories often exhibit multi-periodic features, with variations in start times, arrival times, and sampling frequencies. External factors such as weather, traffic events, and accidents introduce significant spatiotemporal fluctuations, further complicating accurate prediction. While previous work (e.g., OD-PROPHET [20]) addresses the issue of sequence start-time shifts, it does not fully capture the finer spatiotemporal fluctuations and irregularities within local trajectory segments, hindering accurate representation of dynamic travel behavior.
2. **Partial trajectory comprehension:** Many studies assume the availability of complete trajectory data, yet in practice, such data is often incomplete due to factors like low sampling rates, sensor inaccuracies (e.g., GPS obstruction), and privacy protection measures. These issues lead to sparse, low-quality data, which significantly hampers prediction accuracy, particularly in scenarios with missing trajectory segments.
3. **Undifferentiated spatial error treatment:** Traditional cross-entropy loss functions in trajectory prediction treat all prediction errors equally, disregarding the geographic proximity between predicted and actual locations. This coarse-grained approach fails to account for errors that are spatially close, which should be penalized less than errors involving larger distances. This limitation reduces the model’s ability to capture spatial consistency, weakening its overall predictive accuracy and robustness.

These challenges stem directly from the limitations of traditional and deep learning-based methods and highlight the need for more sophisticated approaches to the prediction of travel destinations. To address these issues, this paper proposes an improved method that integrates multi-task learning, a multi-trajectory subsequence alignment attention mechanism, and a spatial consistency-constrained cross-entropy loss function, significantly enhancing prediction performance in complex scenarios. Unlike existing works that treat data sparsity and noise as independent issues [16,17], our proposed MTSA-SC framework jointly optimizes trajectory completion and prediction through multi-task learning—enabling the model to recover critical mobility semantics from incomplete inputs (e.g., reconstructing hidden detours from sparse GPS pings). This contrasts with TrajBERT’s linear interpolation approach, which enforces uniform sampling intervals and distorts temporal dynamics, as well as OD-PROPHET’s rigid architecture [20] that fails to capture local spatiotemporal fluctuations.

Furthermore, the proposed mechanism introduces behavioral pattern disentanglement through dynamic template matching, distinguishing intrinsic mobility semantics from transient noise via cross-referenced trajectory prototypes. It models spontaneous fluctuations via attention-driven spatiotemporal morphing operators that align localized deviations with historical patterns, overcoming linear interpolation limits. This dual capability enables adaptive handling of heterogeneous signal degradation while preserving path diversity through nonlinear trajectory completion.The main contributions of this paper are summarized as follows:

- **Trajectory completion and prediction co-optimization**. Our multi-task learning architecture synergizes trajectory restoration and destination prediction through: (1) Shared encoder representations that preserve spatiotemporal semantics between tasks, (2) Complementary training objectives that enforce consistency between reconstructed paths and predicted destinations. By dynamically capturing mobility patterns from fragmented observations, the framework simultaneously reconstructs missing segments (e.g., low-frequency sampling gaps) and infers destinations through mutual feature enhancement,overcoming the error propagation limitations of sequential processing paradigms.
- **Multi-trajectory subsequence alignment attention**. To tackle the challenges of trajectory asynchrony and local fluctuation, this paper proposes a multi-trajectory subsequence alignment attention mechanism. This mechanism extracts multi-scale local trajectory segments using a sliding window, constructs a diverse alignment template library, and dynamically matches local trajectory features with lightweight convolutional kernels, enabling the model to adaptively capture sudden fluctuations (e.g., detours, congestion). By integrating global and local information through cross-sequence attention weight fusion, the model improves adaptability to complex travel scenarios.
- **GeoDistance-adaptive loss**. This study designs a GeoDistance-Adaptive Loss framework that fundamentally redefines the evaluation of spatial errors in location prediction. By integrating an exponential spatial decay operator that dynamically modulates penalty intensity based on Euclidean distance errors, the method establishes nonlinear mapping where penalties progressively amplify for distant deviations while mitigating adjacent discrepancies. The core mechanism embeds geographical semantics through dual adaptation: distance-sensitive gradient scaling during backpropagation and magnitude-aware error stratification, which jointly enforce spatial-topological consistency.
- **Systematic experimental validation**. Experiments on two large-scale trajectory datasets from Shenzhen and Xiamen demonstrate that our proposed method achieves prediction accuracies of recall rates of 0.722 and 0.6, respectively, in complete and sparse trajectory scenarios, which demonstrate improvements of 0.115 and 0.074 over the best baseline methods (TrajBERT). The datasets and code generated during the current study are available in the GitHub repository: https://github.com/DanLuo-work/MTSA-SC.

## Related work

Individual mobility prediction serves as a key domain in spatiotemporal data mining, encompassing a range of tasks from micro-level POI (Point of Interest) prediction to macro-level travel path modeling. Current research primarily focuses on two core directions: Next POI prediction and destination prediction. Below, we discuss their respective advancements and common challenges.

### Next POI prediction

Next POI prediction aims to infer the future interest points a user may visit based on their historical trajectory, serving as a core technology for recommendation systems. Early studies relied on classical statistical models, such as MLE (maximum likelihood estimation) [21] and EM (Expectation Maximization) [22] , which predict future values by weighted averaging historical observations. While these methods are computationally efficient, they struggle to model complex mobility patterns and capture the dynamic and diverse nature of user behavior.

The introduction of Collaborative Filtering (CF) and Matrix Factorization (MF) techniques partially alleviated these issues. User-based CF methods [23] enhance recommendation accuracy by leveraging similarities in group behavior, while MF methods [24] incorporate geographic and social features to optimize user-POI interaction modeling. However, both approaches face challenges related to data sparsity and diversity: CF struggles with inaccurate similarity measurements under sparse data, and MF is prone to overfitting due to high-dimensional sparsity, while both neglect the dynamic impact of spatiotemporal contexts on user preferences.

Probabilistic models further improved the ability to model complex behaviors. The Gaussian Mixture Model (GMM) [25] captures user mobility patterns through weighted sums of multiple Gaussian distributions, while the Hidden Markov Model (HMM) [26] models trajectory temporal dependencies using state transition sequences. However, GMM lacks temporal modeling capabilities and is poorly suited for high-dimensional sparse data; HMM is constrained by the Markov assumption, limiting its ability to capture long-term dependencies and nonlinear dynamics. Both models rely on strong distributional assumptions, leading to parameter estimation challenges and difficulty adapting to complex scenarios.

Deep learning methods have advanced performance through feature fusion and attention mechanisms. VANext [27] employs spatiotemporal attention to model periodic behaviors, while MMPOI [28] integrates multimodal data (such as geographic information, social networks, and preference profiles) to enhance contextual awareness. Huang *et al*. [29] addressed the issue of neglecting discrete time slot preferences in Next POI recommendations by proposing a hierarchical mobility tree structure approach. This method constructs multi-granularity time slot nodes to differentiate user preferences across different time periods and introduces a Mobility Tree Network (MTNet), designing four-step node interaction operations and multi-task training strategies to enhance prediction performance. TrajRecovery [30] recovers city-scale vehicle trajectories using traffic cameras via a spatial transfer probabilistic model (STPM) and rule-enhanced generation. STPM probabilistically infers turning behaviors by fusing road topology with driver preferences, while generating continuous trajectories from sparse snapshots. Despite successful real-world deployment, limitations include sensitivity to sparse camera coverage, unverified handling of non-compliant driving behaviors, and error accumulation risks in STPM’s probabilistic chains during traffic fluctuations. CDSTraj [31] leverages semantic-augmented diffusion with lane-aware kinematic constraints to model trajectory uncertainty, enhanced by graph-based attention for vehicle-infrastructure interactions. While achieving high accuracy, its iterative denoising causes computational delays, hindering real-time use, and static semantic dependencies restrict adaptability to dynamic road changes (e.g., temporary closures).

### Destination prediction

Destination prediction infers a user’s final destination based on their current travel trajectory and related contextual information. Traditional statistical methods utilize the probability distribution of historical travel records or clustering techniques [9,10], offering simple and intuitive solutions but struggling with data sparsity and complex behavioral patterns.

In recent years, deep learning models have become dominant in destination prediction. Recurrent Neural Networks (RNN) and their variants, such as Long Short-Term Memory (LSTM) networks [32], are widely applied for time series modeling, significantly improving prediction accuracy by capturing trajectory temporal dependencies. However, LSTM performance weakens when dealing with sparse or incomplete long sequences due to data complexity. Additionally, Temporal Graph Neural Networks (TGNN) [33] enhance destination prediction by integrating graph structures with temporal interaction information but face computational efficiency challenges in large-scale graph scenarios. These studies provide new insights into individual mobility prediction but require further optimization in handling data sparsity and local volatility modeling.

Various attention-based models [15,34,35]have also been introduced to destination prediction tasks, leveraging their global modeling capabilities to capture dependencies among key points in trajectories. However, most Transformer models assume complete and high-quality trajectory data, failing to adequately address the impact of data sparsity and noise on prediction accuracy. Despite these limitations, deep learning methods offer robust support for modeling complex trajectory patterns and provide critical directions for future research. RuleKG-MobiPre [36] employs a rule-enhanced knowledge graph framework to model mobility patterns by integrating multi-hop relational path semantics and user-specific hyperplanes. It extracts logical rules from relational paths to capture long-term dependencies (e.g., daily routines) while embedding user characteristics into entity-relation hyperplanes. Although enhancing interpretability, it faces scalability challenges in rule mining for large-scale KGs and unverified adaptability to real-time trajectory dynamics. RouPID [37] addresses route uncertainty in instant delivery via a 3-modal fusion network combining BLE encounters, site metadata, and GPS trajectories. An edge-enhanced relational graph attention network models global decision factors (e.g., order constraints), yet its performance depends on precise sensor synchronization and lacks validation in heterogeneous delivery ecosystems with infrastructure disparities (e.g., sparse BLE coverage).

Despite significant advancements in individual mobility prediction technologies, multiple challenges remain. Traditional statistical and probabilistic models are computationally efficient but struggle to model complex behaviors and long-term dependencies. CF and MF are constrained by data sparsity and overlook spatiotemporal dynamics. While deep learning methods have improved performance, they still fall short in handling sparse data and capturing local volatility, often relying excessively on data integrity.

This paper proposes a method combining multi-task learning, trajectory subsequence alignment attention mechanisms, and spatial consistency constraint loss functions to address these issues and achieve more precise mobility predictions. By enhancing the model’s adaptability to sparse data and its ability to capture local volatility while improving spatial consistency in prediction results, this approach provides a more reliable solution for individual mobility prediction.

## Preliminary

Unlike traditional methods that assume uniformly spaced time intervals, our approach models user activities with irregular and non-uniform intervals, providing a more precise representation of arrival and departure times. This allows for a more realistic and flexible modeling of user behavior, which is crucial for accurate destination prediction.

We define the sets of users, location nodes, and environmental contexts as 𝒰, 𝒱, and 𝒞, respectively. The location nodes, representing urban street blocks, are defined as $𝒱=\{v_{1},v_{2},\ldots ,v_{N}\}$, where *N* is the total number of blocks.

**Definition 1 (Sparse Prefi Trajectory Set).** *For each user u∈𝒰, the trajectory ${𝒳}_{u}^{v}$ is a sequence of tuples ${𝒳}_{u}^{v}=\{(v_{i},t_{v_{i}})|i=1,2,\ldots ,M\}$, where each tuple $\left(v_{i},t_{v_{i}}\right)$ represents a visit of user u to location $v_{i}$ during a time interval $t_{v_{i}}$. The time interval $t_{v_{i}}$ is modeled as $t_{v_{i}}=(t_{a},\Delta t_{a},t_{b},\Delta t_{b},t_{w})$. Here, t_a_ and t_b_ are the arrival and departure time intervals, respectively; $\Delta t_{a}$ and $\Delta t_{b}$ are the specific time shifts within t_a_ and t_b_; and t_w_ represents the day of the week (e.g., Monday, Tuesday, etc.) corresponding to the visit.*

For instance, with 15-minute time intervals, a user arrives at location $v_{i}$ on Monday at 11:32:21 and departs at 12:13:47 can be represented as $t_{v_{i}}=(46,141,48,827,1)$. Here, *t*_*a*_ = 46 and *t*_*b*_ = 48 are the arrival and departure interval indices, respectively; $\Delta t_{a}=141$ and $\Delta t_{b}=827$ are the offsets (in seconds) from the interval starts; and *t*_*w*_ = 1 indicates the visit occurred on Monday.

**Definition 2 (Environmental Context Set).** *For each location point $v_{i}$ in the trajectory ${𝒳}_{u}^{v}$, the environmental context sequence ${𝒳}_{u}^{c}$ associates a d_c_-dimensional feature vector $c_{i}\in {\mathbb{R}}^{d}$. Each vector c_i_ encodes spatiotemporal information, such as road network topology, points of interest (POIs), and weather conditions, relevant to the location $v_{i}$ at the corresponding time interval. The sequence is denoted as ${𝒳}_{u}^{c}=\{c_{1},c_{2},\ldots ,c_{M}\}$*.

Given a prefix trajectory ${𝒳}_{u}^{v}=\{(v_{1},t_{v_{1}}),(v_{2},t_{v_{2}}),\ldots ,(v_{k},t_{v_{k}})\}$ of a trip by user *u*, along with the corresponding environmental context sequence ${𝒳}_{u}^{c}$, the goal of our work is to predict the destination $v_{k+1}$ of the trip, where $v_{k+1}$ is the final location in the user’s movement sequence.

## The MTSA-SC model

**Fig 1:**
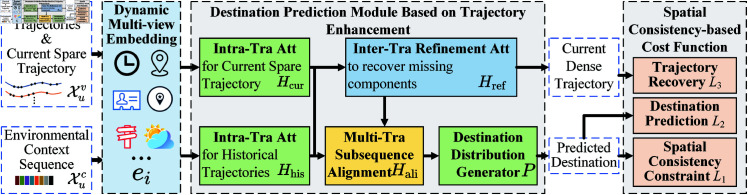
The overview of MTSA-SC.

As illustrated in Fig 1, the MTSA-SC model integrates multiple advanced techniques to address the challenges of sparse and incomplete trajectory data in destination prediction. The **dynamic multi-view embedding** mechanism serves as the initial processing stage, encoding both historical and current sparse trajectory data along with environmental context. It transforms raw trajectory data into dense vectors, capturing complex spatiotemporal dependencies and leveraging multi-source contextual information to enhance feature expressiveness. Following the embedding stage, the trajectory refinement module incorporates a series of attention-based mechanisms to enhance data continuity and completeness. The **intra-trajectory attention** mechanism captures local spatiotemporal dependencies in both historical and current trajectories, while the **inter-trajectory refinement** attention improves trajectory continuity by recovering missing components. To ensure trajectory alignment at multiple scales, the **multi-trajectory subsequence alignment** attention mechanism fine-tunes segment matching, reducing inconsistencies and improving the fidelity of travel pattern representation. With refined trajectory information, the **destination distribution generator** produces a probability distribution over potential destinations. To further enhance the reliability of predictions, a **spatial consistency-based cost function** is introduced, which jointly optimizes trajectory recovery, destination prediction, and spatial coherence. By dynamically adjusting the penalty for spatial deviations, this function ensures that predicted destinations align with plausible movement patterns, thereby improving overall prediction robustness. By leveraging multi-task learning, the MTSA-SC model simultaneously addresses trajectory completion and destination prediction, ensures that both global movement trends and fine-grained trajectory variations are effectively captured, allowing it to maintain high performance even in scenarios with sparse and incomplete data.

### Dynamic multi-view embedding

Human mobility sequences contain abundant spatiotemporal information, but traditional methods often struggle to capture the complex and diverse patterns within sparse trajectories. To address this limitation, we propose a multi-view embedding framework that integrates multiple perspectives, including spatiotemporal embedding, personalized context embedding, and environmental context embedding.

#### Spatio-temporal embedding.

To effectively model the spatiotemporal distribution of sparse trajectories, we separately embed spatial and temporal features into dense vector representations.

**Spatial embedding:** We first apply one-hot encoding to transform each location node $v_{i}$ in the trajectory ${𝒳}_{u}^{v}$ into an *N*-dimensional sparse vector, uniquely identifying each node in the high dimensional space. Subsequently, through a fully connected layer with a weight matrix $W_{s}\in {\mathbb{R}}^{N\times d_{s}}\left(d_{s}\ll N\right)$, the sparse vector is converted into a *d*_*s*_-dimensional dense vector $D_{s}\left(\nu_{i}\right)\in {\mathbb{R}}^{d_{s}}$, achieving the spatial embedding of the location node. To dynamically adjust the connection weights between nodes and capture spatial relationships, we adopt a graph embedding method to map all nodes in the graph to a low-dimensional vector space, initializing *W*_*s*_. This allows the model to adaptively learn spatial dependencies between street blocks. Additionally, to enhance the model’s position perception of trajectory points, we introduce position encoding. If the embedding dimension *d*_*s*_ is even:

$${PE}_{i,k}=\sin\left(\frac{i}{10000^{\frac{k}{d_{s}}}}\right).$$ (1)

If *d*_*s*_ is odd:

$${PE}_{i,k}=\cos\left(\frac{i}{10000^{\frac{k-1}{d_{s}}}}\right),$$ (2)

Here, *PE*_*i*,*k*_ represents the position encoding of the *i*-th point in the *k*-th dimension of the trajectory. The final spatial feature is obtained by adding the position encoding to the dense representation:

$$D_{s}^{\prime }(v_{i})=D_{s}(v_{i})+PE_{i}.$$ (3)

This design injects explicit sequential information into the model through sinusoidal encoding, preserving trajectory order despite the permutation-invariant nature of self-attention. The varying frequencies of sine and cosine functions across dimensions enable the model to better capture sequential patterns in trajectories.

**Temporal embedding:** To capture the temporal dynamics of user mobility, we design a comprehensive temporal embedding that encodes both discrete time intervals and continuous time shifts. Similar to spatial embedding, we embed temporal interval $t_{v_{i}}=(t_{a},\Delta t_{a},t_{b},\Delta t_{b},t_{w})$ into dense vectors. The arrival time interval *t*_*a*_ and departure time interval *t*_*b*_ are first embedded into dense vectors using learned embeddings, which map each discrete time interval to a low-dimensional space. Next, the time shifts $\Delta t_{a}$ and $\Delta t_{b}$, representing the precise offsets within their respective intervals, are encoded using linear projection to capture fine-grained temporal variations. Additionally, to model weekly periodicity in user behavior, the day of the week *t*_*w*_ is embedded into a dense vector. The final temporal embedding $D_{t}(t_{v_{i}})$ is obtained by concatenating these components, resulting in a rich representation that captures both coarse-grained time intervals and fine-grained temporal details. This enables the model to effectively learn temporal patterns in user mobility, such as daily routines and weekly habits.

The spatiotemporal embedding $D_{st}(v_{i},t_{v_{i}})$ is then formed by combining the spatial and temporal embeddings:

$$D_{st}(v_{i},t_{v_{i}})=[D_{s}^{\prime }(v_{i});D_{t}(t_{v_{i}})].$$ (4)

#### Personalized and environmental context embedding.

To model user-specific behaviors and environmental influences, we jointly embed user IDs and environmental context features. Specifically, we use an embedding layer with a weight matrix $W_{uc}\in {\mathbb{R}}^{(|𝒰|+d_{c})\times d_{uc}}$ (where $d_{uc}\ll |𝒰|+d_{c}$) to concatenate the user’s one-hot sparse vector with the *d*_*c*_-dimensional environmental context feature vector. This is then transformed into a *d*_*uc*_-dimensional dense vector $D_{uc}(u,c_{i})\in {\mathbb{R}}^{d_{uc}}$, capturing both user-specific and environmental features. It compresses the high-dimensional user identity space into a dense semantic subspace to avoid overparameterization, and enables dynamic interaction between user preferences and environmental conditions in a shared latent space.

#### Multi-view fusion.

After obtaining the spatiotemporal embedding $D_{st}(v_{i},t_{v_{i}})$ and the joint personalized and environmental context embedding $D_{uc}(u,c_{i})$, we fuse these multi-view embeddings for each location point of user *u*. Through concatenation, we obtain a comprehensive multi-view fused embedding representation $e_{i}=[D_{st}(v_{i},t_{v_{i}});D_{uc}(u,c_{i})]\in {\mathbb{R}}^{d_{st}+d_{uc}}$, where [;] denotes the concatenation operation.

### Destination prediction framework based on trajectory enhancement

The **Destination Prediction Framework Based on Trajectory Enhancement** improves the quality of current trajectory representations by integrating historical and current trajectory information, enabling more accurate destination prediction. As a multi-task learning framework, it aims to collaboratively optimize multiple tasks, enhancing both trajectory completion and destination prediction. The framework consists of three core submodules: **Intra-Trajectory Attention**, **Inter-Trajectory Refinement**, and **Destination Distribution Generator**.

#### Intra-trajectory attention.

To comprehensively model spatiotemporal dependencies within users’ historical and current trajectories, we employ a multi-head self-attention mechanism that simultaneously captures intra-trajectory dynamics and handles sparse observations.

First, each location point $v_{i}$ with its multi-view embedding $e_{i}\in {\mathbb{R}}^{d}$ undergoes three key transformations to generate query (*Q*), key (*K*), and value (*V*) representations through learned projection matrices $W_{Q}^{s1},W_{K}^{s1},W_{V}^{s1}\in {\mathbb{R}}^{d\times d}$. Then, the attention mechanism computes pairwise relevance scores between trajectory points using scaled dot-product:

$$\alpha_{i,j}=\frac{\langle W_{Q}^{s1}e_{i},W_{K}^{s1}e_{j}\rangle }{\sqrt{d_{h}}}$$ (5)

where *d*_*h*_ = *d*/*L* denotes the dimension per attention head, and *L* is the total number of attention heads. To address missing data, we apply a binary mask that nullifies contributions from unobserved positions:

$$\alpha_{ij}=\left\{ \begin{array}{cc} \alpha_{ij} & \text{if}\;v_{j}\text{ is observed}\\ -\infty & \text{otherwise} \end{array}\right.$$ (6)

Attention weights are then normalized through softmax:

$$\beta_{ij}=\frac{\exp(\alpha_{ij})}{\sum_{m=1}^{M}\exp(\alpha_{im})}$$ (7)

Each attention head produces context-aware representations through weighted aggregation:

$$h_{i}^{\left(l\right)}=\sum_{j=1}^{M}\beta_{ij}^{\left(l\right)}W_{V}^{s1(l)}e_{j}$$ (8)

where l∈{1,...,L} indexes the attention heads. All heads’ outputs are concatenated and linearly projected to form the final representation:

$$e_{i}^{\prime }=W_{O}^{s1}\left[h_{i}^{\left(1\right)}\|\cdots \|h_{i}^{\left(L\right)}\right]$$ (9)

where $W_{O}^{s1}\in {\mathbb{R}}^{d\times d}$ is the output projection matrix and ‖ denotes concatenation. The complete trajectory representation $H_{\text{tra}}=[e_{1}^{\prime },...,e_{M}^{\prime }]\in {\mathbb{R}}^{M\times d}$ preserves sequential patterns while mitigating sparse observation impacts through two innovations: 1) Adaptive attention masking that dynamically excludes missing positions, and 2) Multi-scale pattern discovery enabled by independent projection matrices per head.

#### Inter-trajectory refinement.

Based on multi-head self-attention-based feature representations of historical and current trajectories, we propose an inter-trajectory refinement attention mechanism to address data sparsity in the current trajectory by leveraging historical trajectory information to refine and recover missing components, thereby enhancing its completeness and quality for better downstream predictions.

Let $H_{\text{his}}=[e_{1}^{\prime },e_{2}^{\prime },\ldots ,e_{M}^{\prime }]\in {\mathbb{R}}^{M\times d}$ denote the feature representation of the historical trajectory and $H_{\text{cur}}=[e_{1}^{\prime },e_{2}^{\prime },\ldots ,e_{N}^{\prime }]\in {\mathbb{R}}^{N\times d}$ represent the feature representation of the current trajectory. First, for each node $v_{i}$ in the current trajectory with its embedded representation $e_{i}^{\prime }$ and each node $v_{j}$ in the historical trajectory with its embedded representation $e_{j}^{\prime }$, we compute the cross-correlation coefficient $\gamma_{e_{i}^{\prime },e_{j}^{\prime }}$ as:

$$\gamma_{e_{i}^{\prime },e_{j}^{\prime }}=\langle W_{Q}^{c1}e_{i}^{\prime },W_{K}^{c1}e_{j}^{\prime }\rangle ,$$ (10)

where $W_{Q}^{c1},W_{K}^{c1}\in {\mathbb{R}}^{d\times d}$ are learnable cross-transformation matrices and ⟨·,·⟩ denotes the inner product operation. Then, based on the cross-correlation coefficients, we compute the cross-attention scores $\delta_{i,j}$ using a softmax normalization:

$$\delta_{i,j}=\frac{\exp(\gamma_{e_{i}^{\prime },e_{j}^{\prime }})}{\sum_{k=1}^{M}\exp(\gamma_{e_{i}^{\prime },e_{k}^{\prime }})}.$$ (11)

Here, $\delta_{i,j}$ quantifies the relevance between the *i*-th node of the current trajectory and the *j*-th node of the historical trajectory. A higher score indicates stronger alignment and greater potential for information transfer. Using the computed cross-attention scores, we generate a refined representation $e_{i}^{r}$ for each node $v_{i}$ in the current trajectory:

$$e_{i}^{r}=\sum_{j=1}^{M}\delta_{i,j}W_{V}^{c1}e_{j}^{\prime },$$ (12)

where $W_{V}^{c1}\in {\mathbb{R}}^{d\times d}$ is the cross-value transformation matrix. This step effectively aggregates information from the historical trajectory to compensate for missing or sparse data in the current trajectory. Finally, we combine the refined representation $e_{i}^{r}$ with the original representation $e_{i}^{\prime }$ of the current trajectory node to produce an enhanced and recovered representation:

$$e_{i}^{*}=\omega e_{i}^{r}+(1-\omega )e_{i}^{\prime },$$ (13)

where ω∈[0,1] is a learnable weight that balances the contributions of the refined and original information, and the complete trajectory representation is denoted as $H_{\text{ref}}=\left[e_{1}^{*},e_{2}^{*},\ldots ,e_{N}^{*}\right]\in {\mathbb{R}}^{N\times d}$. This fusion ensures that the model retains the unique characteristics of the current trajectory while benefiting from historical context.

With the output of the Inter-Trajectory Refinement Attention mechanism, $H_{\text{ref}}$, we input the complete representation of the current trajectory into the Sub-Trajectory Alignment module for analyzing associations between current and historical trajectories based on multi-trajectory subsequence alignment attention mechanisms (see Sect), to further uncover potential associations between historical and current trajectories and obtain information-enhanced complete features of the current prefix trajectory $H_{\text{ali}}$.

### Destination distribution generator

After obtaining the complete trajectory feature $H_{\text{ali}}$, we introduce a destination distribution generator to predict the probability distribution of the destination for the current trajectory.

First, we calculate the average representation of the trajectory feature to capture the overall characteristics of the trajectory:

$$H_{\text{ali}}^{\prime }=\frac{\sum_{i=1}^{L_{\text{ali}}}e_{i}}{L_{\text{ali}}}$$ (14)

where $L_{\text{ali}}$ is the length of the user’s trajectory, *e*_*i*_ is the feature of the *i*-th point in the trajectory, $H_{\text{ali}}^{\prime }\in {\mathbb{R}}^{1\times d}$, and *d* represents the dimension of the embedded vector. Then, the average representation $H_{\text{ali}}^{\prime }$ is input into a linear layer for transformation:

$$e_{p}=H_{\text{ali}}^{\prime }W_{l}+b$$ (15)

Finally, after passing through the Softmax function, the predicted probability distribution is obtained:

$$P=\text{Softmax}(e_{p})$$ (16)

where $W_{l}\in {\mathbb{R}}^{d\times N}$ represents the linear transformation matrix, *N* is the number of all possible position points, *b* is the bias vector of the linear transformation, and *P* is the probability distribution over all possible destination points, representing the likelihood of the destination of the current trajectory sequence.

### Multi-trajectory subsequence alignment

After obtaining the output $H_{\text{ref}}=\left[e_{1}^{*},e_{2}^{*},\ldots ,e_{N}^{*}\right]\in {\mathbb{R}}^{N\times d}$ from the inter-trajectory refinement attention, we input the representation into the current-historical trajectory association analysis module based on the multi-trajectory subsequence alignment attention mechanism. Unlike the previous method proposed in [20] that only performs translation alignment based on global trajectory correlations, this mechanism focuses on fine-grained segment alignment. By analyzing local trajectory dynamics and employing sliding windows alongside convolution operations, the mechanism enhances its ability to manage intricate trajectory patterns.

#### Convolution Kernel construction.

The multi-trajectory alignment attention mechanism is based on a sliding window approach, with the first step being the construction of multiple 2D convolution kernels. Using different convolution kernels (each corresponding to a distinct subsequence template), the model can analyze trajectories from various angles. These kernels capture complex spatiotemporal dependencies between trajectories, with each kernel representing a specific subsequence matching pattern, thus increasing subsequence diversity and improving the model’s ability to handle trajectory variations.

**Fig 2:**
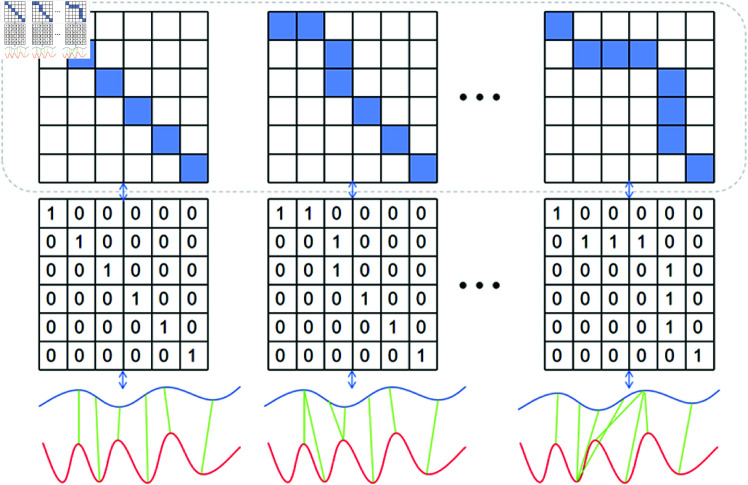
Different convolution kernels for trajectory alignment template.

As shown in Fig 2, a zero-matrix $K\in {\mathbb{R}}^{n\times m}$ is defined, where *n* and *m* are predefined dimensions. The top-left element *K*_11_ is set to 1. Then, starting from *K*_11_, an adjacent element (to the right, downward, or bottom-right) is set to 1. For example, when at *K*_11_, we can set one of the elements *K*_12_, *K*_21_, or *K*_22_ to 1. This process continues until the bottom-right element *K*_*nm*_ is reached. These matrices form a cluster of convolution kernels, each corresponding to a different subsequence matching pattern, which are then used in subsequent convolution operations.

#### Multi-trajectory alignment attention based on sliding window.

**Fig 3:**
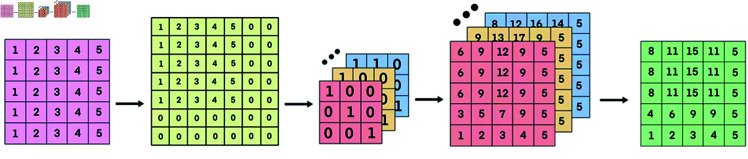
Multi-trajectory alignment attention based on sliding window.

As shown in Fig 3, after the convolution kernels are constructed, the next step is to transform the current trajectory representation $H_{\text{ref}}=\left[e_{1}^{*},e_{2}^{*},\ldots ,e_{N}^{*}\right]$ and historical trajectory representation $H_{\text{his}}=[e_{1}^{\prime },e_{2}^{\prime },\ldots ,e_{M}^{\prime }]$ into query, key, and value matrices:

$$Q_{c}=W_{Q}^{c}H_{\text{ref}},\;K_{c}=W_{K}^{c}H_{\text{his}},\;V_{c}=W_{V}^{c}H_{\text{his}},$$ (17)

where $W_{Q}^{c}\in {\mathbb{R}}^{d\times d}$, $W_{K}^{c}\in {\mathbb{R}}^{d\times d}$, and $W_{V}^{c}\in {\mathbb{R}}^{d\times d}$ are learnable transformation matrices, and *d* represents the dimension of the embedded vector. Then, the initial cross-attention matrix *X* is calculated as follows:

$$X=Q_{c}K_{c}^{T}.$$ (18)

Here, $Q_{c}\in {\mathbb{R}}^{N\times d}$ represents the weighted vector representation matrix of the current trajectory sequence, where *N* is the length of the current trajectory; $K_{c}\in {\mathbb{R}}^{M\times d}$ represents the weighted vector representation matrix of the historical trajectory sequence, where *M* is the length of the historical trajectory. This matrix serves as the foundation for further convolution operations that will help refine the alignment between the current and historical trajectories.

To facilitate subsequent convolution operations, the matrix *X* is padded to ensure it has the appropriate dimensions:

$$X_{\text{pad}}=\text{Pad}(X,\text{row}=m-1,\text{cols}=n-1).$$ (19)

This padding step prepares the matrix for convolution by ensuring that it aligns correctly with the convolution kernels. To efficiently obtain the calculation results of multiple path-matching similarities, we use the pre-constructed two-dimensional convolution kernels $\{{\text{Kernel}}_{i}{\}}_{i=1}^{k}$ (*k* corresponds to the number of exhaustive paths) to perform parallel two-dimensional convolution operations on the padded matrix $X_{\text{pad}}$. This is done in parallel to efficiently compute the results for multiple path-matching similarities:

$$C_{i}=\text{Conv2D}(X_{\text{pad}},{\text{Kernel}}_{i}),\;\forall i\in \{1,2,\cdots ,k\}.$$ (20)

Here, *C*_*i*_ is the result obtained by convolving with the *i*-th two-dimensional convolution kernel ${\text{Kernel}}_{i}$ (corresponding to the *i*-th constructed path). By introducing the sliding window and two-dimensional convolution operations, the model can learn the trajectory data segment by segment, effectively capturing local features and dynamic changes within the trajectory. Specifically, when the trajectory experiences anomalous fluctuations or short-term deviations, the sliding window can automatically focus on these critical regions and extract more representative and discriminative features. This segment-by-segment analysis approach is particularly well-suited to handle local fluctuations in trajectories, enabling the model to be more sensitive to subtle dynamic changes within the trajectory. As a result, it enhances the model’s ability to perceive complex spatiotemporal patterns and improves the accuracy of predictions regarding the trajectory’s future developments.

For the multi-channel results obtained from multiple path-matching calculations, we perform a weighted summation based on matching similarity. To avoid issues such as gradient vanishing or explosion, the results must first be scaled before the weighted summation is calculated, as shown below:

$$\text{Sum}[r,c]=\frac{\sum_{i=1}^{k}w_{i}[r,c]*C_{i}[r,c]}{\sqrt{d*(m^{2}+n^{2})}}.$$ (21)

Here, the weight *w*_*i*_*[r*,*c]* is computed as:

$$w_{i}[r,c]=\frac{\exp(C_{i}[r,c])}{\sum_{j=1}^{k}\exp(C_{j}[r,c])},\;\forall i\in \{1,2,\cdots ,k\},$$ (22)

where Sum[r,c] represents the element in the *r*-th row and *c*-th column of the Sum matrix, and *d* represents the dimension of the embedded vector.

Once the weighted summation is complete, the next step involves normalizing the result to obtain the attention scores:

Score=Softmax(Sum) (23)

These attention scores are then used to generate the attention output representation for each node *i*:

$${\bar{e}}_{i}=\text{Att}(Q,K,V)=\sum_{j=1}^{M}\text{Score}[i,j]W_{V}^{s2}e_{v_{j}}^{L}$$ (24)

where $W_{V}^{s2}\in {\mathbb{R}}^{d\times d}$ is the transformation matrix for the value *V* (i.e., $H_{\text{his}}$). Through the weighted summation and normalization of the multi-channel results, the model dynamically adjusts the contribution of each subsequence, allowing it to capture the local fluctuations of the trajectory with greater precision. The similarity between subsequences is reflected in the weight adjustment, ensuring that critical local features have a greater impact on the final result. By optimizing the weights based on the local features, the model ensures that the most important characteristics dominate the final prediction. This dynamic adjustment enhances the model’s adaptability, enabling it to make more accurate predictions even as the trajectory characteristics vary, thereby improving its response to complex changes in the trajectory.

Finally, to capture different features between nodes, we can use multiple attention heads simultaneously to get ${\tilde{e}}_{i}$, and then concatenate the outputs of each head to obtain the final node representation. Finally, the complete trajectory feature is $H_{\text{ali}}=\{{\tilde{e}}_{1},{\tilde{e}}_{2},\cdots ,{\tilde{e}}_{L}\}$, where *L* represents the length of the trajectory.

### Spatial consistency-based cost function

To more effectively explore and leverage the model’s ability in spatial learning, we design a cross-entropy loss function based on spatial consistency constraint. On the basis of the traditional cross-entropy loss, this loss function introduces a spatial distance constraint mechanism, which imposes lower penalties on inaccurate predictions for geographically close locations. This approach enhances the spatial consistency of the model. By explicitly incorporating spatial position relationships into the loss calculation, the model is guided to pay more attention to the prediction results of geographically close locations, thereby further improving the refined prediction of travel patterns.

The total loss function based on spatial consistency constraint designed in this paper is defined as:

$$L=\beta_{1}*L_{1}+\beta_{2}*L_{2}+\beta_{3}*L_{3}$$ (25)

where $\beta_{1}$, $\beta_{2}$, and $\beta_{3}$ are the weight hyperparameters of the loss function, *L*_1_ is the cross-entropy loss based on spatial consistency constraint, *L*_2_ is the cross-entropy loss of the trajectory prediction task, and *L*_3_ is the cross-entropy loss of the trajectory recovery task.

The spatial consistency constraint loss *L*_1_ is defined as:

$$L_{1}=-\sum_{i=1}^{N}\sum_{j=1}^{M}\delta_{i,j}\log P\left({\text{loc}}_{i,j}|\theta \right)$$ (26)

where *N* represents the number of trajectories, *M* represents the total number of location points in the road network, $P\left({\text{loc}}_{i,j}|\theta \right)$ represents the probability that the model predicts the location point ${\text{loc}}_{i,j}$, and $\delta_{i,j}$ is the weighting coefficient, defined as:

$$\delta_{i,j}=\left(1-P\left({\text{loc}}_{i,j}|\theta \right)\right)*\left(1-\omega \left({\text{loc}}_{i,j}\right)\right)$$ (27)

$\omega \left({\text{loc}}_{i,j}\right)$ is the spatial distance weight, defined as follows:

$$\omega \left({\text{loc}}_{i,j}\right)=\left\{ \begin{array}{cc} 1, & \text{if}\;\text{dis}\left[{\text{loc}}_{i}\right]\left[{\text{loc}}_{j}\right]=0\\ \frac{\exp\left(-\text{dis}\left[{\text{loc}}_{i}\right]\left[{\text{loc}}_{j}\right]\right)}{\sum_{k=1}^{M}\exp\left(-\text{dis}\left[{\text{loc}}_{i}\right]\left[{\text{loc}}_{k}\right]\right)}, & 0<\text{dis}\left[{\text{loc}}_{i}\right]\left[{\text{loc}}_{j}\right]<\gamma \\ 0, & \text{if}\;\text{dis}\left[{\text{loc}}_{i}\right]\left[{\text{loc}}_{j}\right]\geq \gamma \end{array}\right.$$ (28)

where $\text{dis}\left[{\text{loc}}_{i}\right]\left[{\text{loc}}_{j}\right]$ is the geodesic distance between location points *i* and *j* (calculated from longitude and latitude), and γ is the distance threshold hyperparameter.

Eq 26 calculates the weighted cross-entropy loss for all location points in the trajectory prediction task by introducing the spatial consistency constraint. When the geographical distance between the predicted location and the true location is short, the weight $\omega \left({\text{loc}}_{i,j}\right)$ reduces the penalty for errors, making the model more fault-tolerant in handling geographically close predictions. Conversely, when the geographical distance between the predicted location and the true location is long, the loss function increases the penalty for errors, strengthening the model’s learning of spatial consistency.

In general, the loss function based on spatial consistency constraint effectively improves the model’s performance in geographically close predictions by introducing spatial position relationships. Its design enhances the model’s ability to learn spatial features and improves the accuracy of complex travel pattern predictions, providing valuable theoretical support and practical application value for spatiotemporal data mining.

## Experiments and analysis

### Experimental setups

#### Evaluation data.

**Fig 4:**
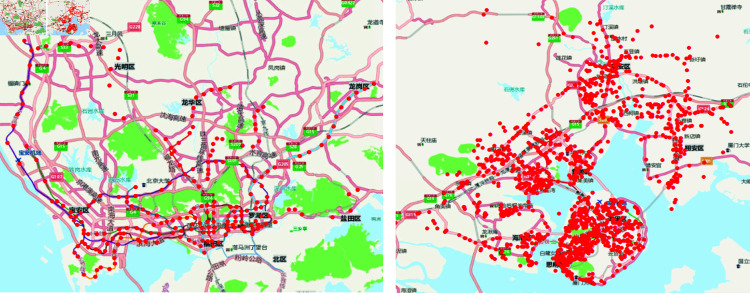
Spatial distribution of rail transit and bus system in Shenzhen and Xiamen.

Our evaluation is based on two real-world datasets, as shown in Table 1 and visualized in Fig 4. The datasets cover rail transit information from Shenzhen and bus system data from Xiamen, both spanning from March 1st, 2022, to May 31st, 2022. The Shenzhen dataset includes data from 2013 users, 5192 points of interest (POIs), and 725,251 trajectory records. The Xiamen dataset contains information from 4955 users, 6014 POIs, and 297,155 trajectory records. For dataset partitioning, we sort each user’s samples chronologically. Starting from the second day, we use the first 70% of the data as the training set (ensuring each sample has at least one day of historical data), the next 10% as the validation set, and the remaining 20% as the test set.

**Table 1:** Experimental dataset description.

Dataset	Transportation mode	# Users	# OD records	# Locations
Shenzhen	subway	10,535	725,251	5,192
Xiamen	bus	4,955	297,155	6,014

#### Model parameter settings.

To ensure a fair comparison among models, all baselines were deployed on a server equipped with a NVIDIA V100 GPU. For optimization, we employed the Adam optimizer with default parameter settings, an initial learning rate of 0.0005, and a learning rate decay threshold of $9\times 10^{-7}$, at which point the training process was terminated. The training for the Shenzhen dataset ran for approximately 30 epochs, and for the Xiamen dataset, it continued for about 40 epochs. The maximum length for trajectory sequences was set to 12, with a maximum of 21 historical samples considered for each user. Keeping these shared hyperparameters constant, we further optimized the unique hyperparameters of each model through experiments. In our model, the optimal embedding dimension was set to 256, the best number of heads for the multi-head attention mechanism was chosen as 4, and the dimension of the convolution kernel was 3.

#### Performance metrics.

Two metrics are adopted to evaluate the performance of destination prediction. The first metric is the **Error Distance Score (EDS)**, which measures the Haversine distance between the predicted destination and the true location. The second metric is **Recall@k**, which calculates the recall rate of the top 1, top 5, and top 10 predicted destination points for the next trip. A lower EDS and a higher Recall@k indicate better prediction performance.

#### Baselines.

To evaluate the accuracy of our prediction model, we compared the proposed model with several state-of-the-art methods:

- RF [38]: A trajectory prediction scheme based on the random forest. It takes multiple features of position points as input, including position coordinates, time attributes, and features related to the surrounding environment. By constructing multiple decision trees to form a random forest structure, it learns patterns from different trajectory scenarios to predict the trajectory direction and subsequent position information.
- FPMC [39]: A next-location recommendation scheme based on the Markov model. It treats all visited locations as states. Using Markov chain theory, it abstracts each location in the trajectory into different states, analyzes the transition relationships between states through a factorization model, and predicts the next possible location based on the current state by mining transition probabilities between locations.
- STRNN [40]: A scheme that predicts the next location using Recurrent Neural Networks (RNN). This method leverages the time-series characteristics and spatial location information of trajectory data. Using the inherent structure of RNNs, it processes the spatiotemporal information of each position point sequentially over time, integrating sequential and spatial features to predict subsequent locations.
- DeepMove [17]: A scheme for next-location prediction using RNN with an attention mechanism. It integrates an attention mechanism into the conventional RNN architecture. When processing trajectory data, the attention mechanism dynamically assigns weights based on the importance of different time and position information across the entire trajectory, focusing on the key information that contributes to predicting the next location.
- STGN [41]: A scheme for predicting the next point of interest (POI) using RNN with time-interval and distance-interval gating. It employs RNN as the base framework, enhanced by specially designed gating mechanisms that control the flow of time-interval and distance-interval information within the network, assisting in the prediction of the next POI based on trajectory data.
- AttnMove [42]: A trajectory recovery scheme that utilizes an attention mechanism. Focusing on the core task of trajectory recovery, it processes existing trajectory segments and related features. The attention mechanism assigns appropriate weights, concentrating on the most relevant information to restore the missing parts of the trajectory based on available data.
- LSPTM [43]: A trajectory prediction scheme based on the Transformer model. It uses the unique multi-head attention mechanism in the Transformer architecture to capture the dependencies between different positions in the trajectory. By encoding the position sequence information, it effectively handles the complex correlations of trajectory data in the time and space dimensions to predict subsequent positions in the trajectory.
- TrajBERT [16]: A trajectory representation scheme inspired by Bidirectional Encoder Representations from Transformers (BERT). It performs bidirectional encoding on trajectory data, learning an encoding representation for each position and its contextual information in the trajectory. This representation is versatile, suitable for tasks like trajectory classification, similarity measurement between trajectories, and predicting future positions based on trajectory data.

#### Impact of factors.

To assess the impact on the robustness and generalization ability of MTSA-SC and explore its performance under sparse data conditions, we divided the experimental dataset into four equal parts. The first part contained the complete, unchanged trajectory data. In the second part, we simulated a 25% trajectory missing rate; in the third part, a 50% missing rate; and in the fourth part, a 75% missing rate. This setup allowed us to observe the model’s performance under varying levels of data sparsity.

### Evaluation results

#### Baseline performance comparison.

The overall comparison is presented in Table 2. MTSA-SC consistently outperforms all alternative methods in terms of Recall@k and EDS (m) across the two real-world datasets, Shenzhen and Xiamen, which feature different transportation modes. The specific observations are as follows:

**Table 2:** Performance comparison of destination prediction with baselines on two datasets.

Dataset	Shenzhen				Xiamen			
Baseline	Recall@1	Recall@5	Recall@10	EDS (m)	Recall@1	Recall@5	Recall@10	EDS (m)
RF	0.110	0.310	0.432	6608.84	0.109	0.303	0.428	6644.30
FPMC	0.212	0.532	0.646	5938.24	0.184	0.482	0.601	5492.38
STRNN	0.470	0.775	0.804	4262.04	0.413	0.671	0.762	4903.85
STGN	0.466	0.786	0.836	4330.38	0.421	0.695	0.770	4674.29
DeepMove	0.478	0.794	0.844	4310.32	0.433	0.701	0.791	4489.62
AttnMove	0.503	0.810	0.858	3835.13	0.490	0.788	0.841	3927.28
LSPTM	0.544	0.825	0.861	3210.52	0.502	0.793	0.867	3524.10
TrajBert	0.607	0.834	0.897	2135.18	0.526	0.810	0.886	3320.45
MTSA-SC	0.722	0.883	0.915	1490.23	0.600	0.840	0.895	2433.30

**Fig 5:**
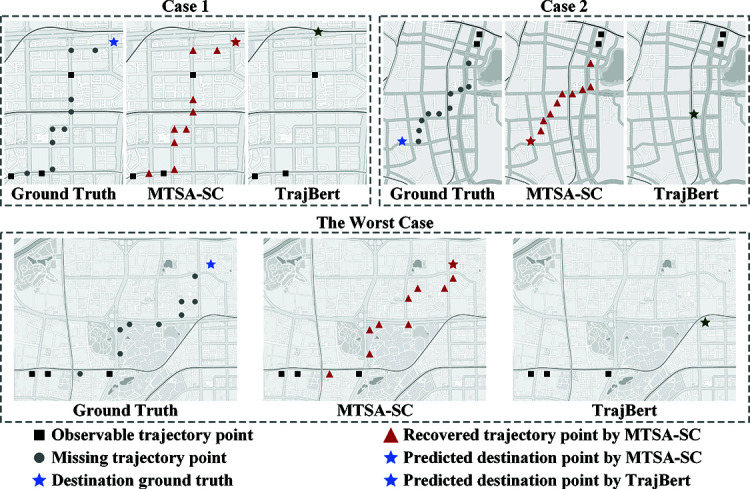
Visualization of destination prediction by MTSA-SC and TrajBERT in two representative cases and the worst case.

- RF-based method: The decision-tree ensemble method (RF) lacks the ability to process sequential data and fails to capture long-term dependencies and temporal information inherent in trajectory data. This limitation becomes particularly evident with trajectory data, which exhibits clear time-sequential patterns, leading to suboptimal performance. On the Shenzhen dataset, Recall@1 was 0.110, Recall@5 was 0.310, Recall@10 was 0.432, and EDS (m) was as high as 6608.84. On the Xiamen dataset, Recall@1 was 0.109, Recall@5 was 0.303, Recall@10 was 0.428, and EDS (m) was 6644.30. In contrast, MTSA-SC improved Recall@1, Recall@5, and Recall@10 by 5.56 times, 1.85 times, and 1.12 times, respectively, compared to RF on the Shenzhen dataset, while reducing EDS (m) by approximately 77.5%. On the Xiamen dataset, Recall@1, Recall@5, and Recall@10 were improved by 4.50 times, 1.77 times, and 1.11 times, respectively, compared to RF, and EDS (m) was reduced by approximately 63.4%.
- FPMC-based method: The Markov model-based method (FPMC) assumes human movement follows the Markov property. However, this simplistic assumption only captures strong position-transfer patterns, neglecting the wealth of other contextual information in trajectory data. This limits its ability to effectively model movement patterns in complex urban road networks. On the Shenzhen dataset, Recall@1 was 0.212, Recall@5 was 0.532, Recall@10 was 0.646, and EDS (m) was 5938.24. On the Xiamen dataset, Recall@1 was 0.184, Recall@5 was 0.482, Recall@10 was 0.601, and EDS (m) was 5492.38. MTSA-SC improved Recall@1, Recall@5, and Recall@10 by 2.40 times, 0.66 times, and 0.42 times, respectively, compared to FPMC on the Shenzhen dataset, and reduced EDS (m) by about 75.0%. On the Xiamen dataset, Recall@1, Recall@5, and Recall@10 were improved by 2.26 times, 0.74 times, and 0.49 times, respectively, compared to FPMC, and EDS (m) was reduced by about 55.7%, highlighting the difficulty FPMC has in effectively handling complex trajectory data and extracting relevant information.
- RNN-based methods (STRNN, STGN): STRNN and STGN excel at processing sequential trajectory data, leveraging the memory units within the RNN structure to process each position sequentially over time. This allows them to capture the dynamic change patterns in the trajectory and the local spatio-temporal correlations between adjacent points. However, RNN-based models face several limitations. First, they suffer from gradient vanishing or explosion when handling long-range dependencies, making it difficult to capture relationships between distant position points in the trajectory. Second, their ability to fuse multi-modal features and external knowledge, such as geographic and weather data, is limited. This restricts their ability to comprehensively understand and predict trajectories in complex real-world scenarios. Compared to the best RNN-based model, STGN, MTSA-SC improved Recall@1, Recall@5, and Recall@10 by 0.55 times, 0.12 times, and 0.10 times, respectively, on the Shenzhen dataset, while reducing EDS (m) by about 65.6%. On the Xiamen dataset, Recall@1, Recall@5, and Recall@10 were improved by 0.43 times, 0.21 times, and 0.16 times, respectively, compared to STGN, and EDS (m) was reduced by about 47.9%.
- Attention-based methods (DeepMove, AttnMove): DeepMove and AttnMove dynamically assign weights to different parts of the input trajectory data, leveraging periodic patterns in historical trajectories to aid in trajectory recovery and prediction. These methods perform well in many cases. However, the best attention-based method, AttnMove, does not outperform MTSA-SC. This is due to coarse-grained historical sparse trajectories, which may not provide significant information gain and could introduce irrelevant co-occurrence relationships. MTSA-SC improved Recall@1, Recall@5, and Recall@10 by 0.44 times, 0.09 times, and 0.07 times, respectively, compared to AttnMove on the Shenzhen dataset, while reducing EDS (m) by about 61.0%. On the Xiamen dataset, Recall@1, Recall@5, and Recall@10 were improved by 0.22 times, 0.07 times, and 0.06 times, respectively, compared to AttnMove, and EDS (m) was reduced by about 38.1%.
- Transformer-based methods (LSPTM, TrajBERT): LSPTM and TrajBERT primarily use the multi-head attention mechanism to capture dependencies between different positions in the trajectory and encode the position sequence. However, they do not specifically address the issue of incomplete trajectory data. In contrast, MTSA-SC employs a specialized trajectory completion and prediction mechanism within a multi-task learning framework, enabling it to handle both missing trajectory completion and trajectory prediction tasks simultaneously. This co-training approach leverages both historical and current trajectory information. When confronted with incomplete trajectory data, MTSA-SC can accurately complete the missing parts and make more precise future trajectory predictions based on the now more complete data. MTSA-SC improved Recall@1, Recall@5, and Recall@10 by 0.19 times, 0.06 times, and 0.02 times, respectively, compared to TrajBERT on the Shenzhen dataset, while reducing EDS (m) by about 30.2%. On the Xiamen dataset, Recall@1, Recall@5, and Recall@10 were improved by 0.14 times, 0.04 times, and 0.01 times, respectively, compared to TrajBERT, and EDS (m) was reduced by about 26.7%. To further visualize the performance gap, Fig 5 compares MTSA-SC with the SOTA baseline (TrajBERT) under three scenarios: trajectories with intermediate verification points (Case 1), short-range sparse movements (Case 2), and long-span complex paths under extreme sparsity (The Worst Case). The multi-task learning structure demonstrates synergistic enhancement between trajectory recovery and destination prediction. The recovered turning points by MTSA-SC provide critical spatiotemporal context, significantly reducing destination deviations compared with TrajBERT. Even under severe data incompleteness, the subsequence alignment mechanism preserves coherent path topology while the spatial consistency loss prevents implausible shortcuts that violate physical road constraints.

#### Trajectory sparsity.

**Fig 6:**
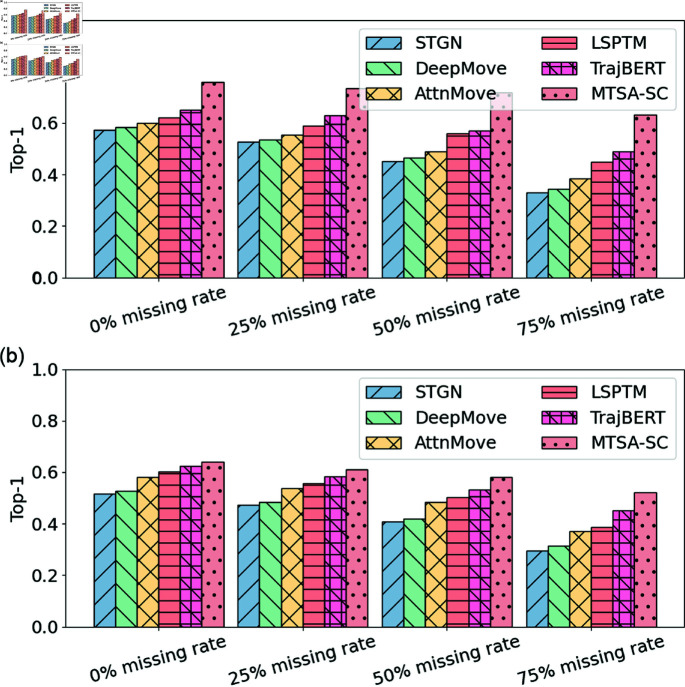
Impact of different trajectory sparsities on the two datasets.

Fig 6 presents the trends of the Top-1 indicators for each model as the trajectory missing rate gradually rises from 0% to 75% on the Shenzhen and Xiamen datasets.

- 0% missing rate: It is clear that at a 0% missing rate, where data is ideal, MTSA-SC already surpasses the other models with a Top-1 value of 0.64039 (Shenzhen) and 0.760 (Xiamen), demonstrating its ability to deliver precise predictions in an optimal environment.
- 25% missing rate: As the missing rate increases to 25%, the data begins to exhibit some sparsity, which impacts each model to different extents. Under this condition, MTSA-SC achieves a Top-1 value of 0.60982 (Shenzhen) and 0.734 (Xiamen), reflecting a relatively minor decline of 4.8% (Shenzhen) and 3.4% (Xiamen) compared to the baseline. This slight reduction shows that MTSA-SC retains robust performance even with a moderate degree of data loss. The model’s multi-task learning structure and its alignment attention mechanism for subsequences enable it to make the most of the available information, which helps to mitigate the challenges posed by incomplete data. On the other hand, other models like STGN and DeepMove experience more significant drops, underlining MTSA-SC’s edge in handling data sparsity.
- 50% missing rate: With a 50% missing rate, data sparsity becomes more pronounced, making the task more difficult for the models. MTSA-SC’s Top-1 value in this scenario is 0.58130 (Shenzhen) and 0.717 (Xiamen), showing a decrease from the 25% missing rate, but still outperforming other approaches. At this point, the gap between MTSA-SC and the rest of the models becomes more noticeable. For instance, the difference between MTSA-SC and STGN reaches 0.1084 (Shenzhen) and 0.0909 (Xiamen), while the gap with DeepMove is 0.09855 (Shenzhen) and 0.0839 (Xiamen). Despite the increased sparsity, MTSA-SC’s performance remains superior, thanks to the enhancements made to its loss function, which integrates spatial consistency constraints. This improvement enables the model to focus on and preserve valuable data points within sparse trajectories, enhancing its prediction accuracy.
- 75% missing rate: When the missing rate reaches 75%, the data becomes highly sparse, presenting a serious challenge for all models. While MTSA-SC’s Top-1 value drops significantly compared to the full dataset, it still achieves the highest performance, with 0.52090 (Shenzhen) and 0.633 (Xiamen). The difference between MTSA-SC and the other models continues to grow, with the gap between MTSA-SC and TrajBERT reaching 0.1139 (Shenzhen) and 0.133 (Xiamen). These results further affirm that MTSA-SC remains the top performer under extreme sparsity conditions. The model’s strong performance in these challenging situations is made possible by its multi-task learning framework, which, combined with the trajectory subsequence alignment attention mechanism and spatial consistency constraint loss, allows it to better leverage sparse trajectory data and sustain high accuracy.

In summary, MTSA-SC consistently leads the performance rankings across different missing rates, especially in environments with high trajectory sparsity. Its exceptional ability to adapt to sparse data can be attributed to the combination of its advanced multi-task learning architecture, attention mechanisms for aligning subsequences, and improved loss function with spatial consistency. These innovations enable MTSA-SC to optimize the use of available data, capture key spatiotemporal information, and ultimately outperform other models, particularly when faced with high sparsity.

#### Ablation experiments.

Table 3 presents the results of the ablation experiments conducted on the Shenzhen and Xiamen datasets to assess the impact of each key component in MTSA-SC. Specifically, we removed three critical modules: the multi-task learning framework (w/o MM), the multi-trajectory subsequence alignment (w/o SSAttn), and the spatial consistency-based cost function (w/o DisLoss).

**Table 3:** Ablation study results.

Dataset	Shenzhen		Xiamen	
Ablation	Recall@1	EDS (m)	Recall@1	EDS (m)
w/o MM	0.685	1854.31	0.564	2841.02
w/o MTSAtt	0.699	1657.86	0.573	2687.65
w/o SCLoss	0.672	2043.47	0.551	3014.57
MTSA-SC	0.722	1490.23	0.600	2433.30

**Fig 7:**
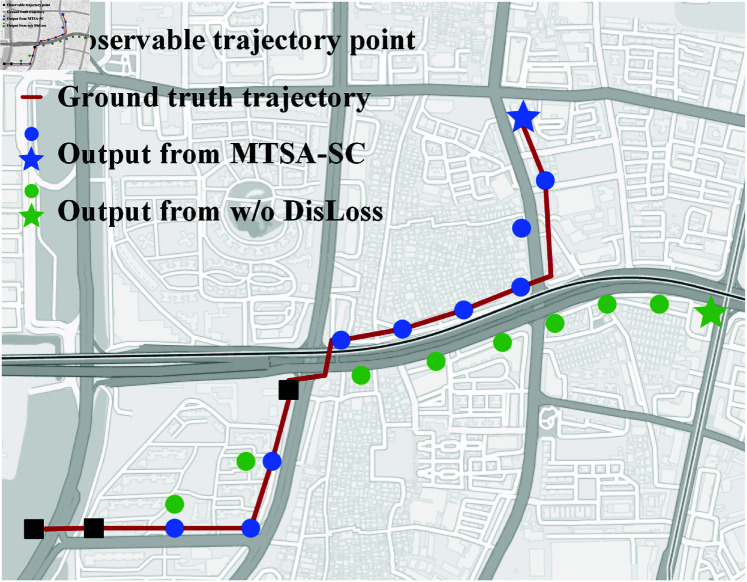
Illustration of destination prediction by MTSA-SC and w/o DisLoss.

- **Removing the multi-task framework (w/o MM):** When the inter-trajectory refinement attention task in multi-task framework was removed, the model experienced a significant decline in both accuracy and precision. In the Shenzhen dataset, the Recall@1 dropped to 0.685, and the EDS (m) increased to 1854.31, with a decrease of 0.037 in Recall@1 and an increase of 364.08 in EDS (m) compared to the complete MTSA-SC. In Xiamen, Recall@1 decreased to 0.564, and EDS (m) increased to 2841.02, a drop of 0.136 in Recall@1 and an increase of 507.02 in EDS (m). This significant performance loss demonstrates that the inter-trajectory refinement attention task is essential for enhancing the model’s ability to refine and complete the current trajectory using historical context.
- **Removing the multi-trajectory subsequence alignment (w/o SSAttn):** Removing the multi-trajectory subsequence alignment led to a reduction in accuracy on both datasets. In Shenzhen, Recall@1 dropped to 0.699, and EDS (m) was 1657.86, showing a 0.023 decrease in Recall@1 and a reduction of 167.63 in EDS (m) compared to the complete MTSA-SC. In Xiamen, Recall@1 dropped to 0.573, and EDS (m) improved to 2687.65, reflecting a 0.027 decrease in Recall@1 and a reduction of 326.92 in EDS (m). The multiple trajectory alignment attention mechanism captures local trajectory changes by constructing alignment templates that allow the model to focus on crucial trajectory patterns. This mechanism enhances the model’s ability to accurately predict subsequent locations by emphasizing important temporal changes. Although removing this mechanism seems to slightly reduce EDS (m), it weakens the model’s ability to align the trajectories correctly, leading to lower accuracy.
- **Removing the spatial consistency-based cost function(w/o DisLoss):** The exclusion of the spatial consistency-based cost function also severely impacted model performance. On Shenzhen, Recall@1 decreased to 0.672, and EDS (m) increased to 2043.47, resulting in a 0.05 drop in Recall@1 and a 553.24 increase in EDS (m) compared to the full model. On Xiamen, Recall@1 dropped to 0.551, and EDS (m) surged to 3014.57, reflecting a 0.049 decrease in Recall@1 and a 523.19 increase in EDS (m). This loss function plays a crucial role in improving the model’s accuracy and spatial precision by penalizing errors based on geographical distance between predicted and true locations. Its removal hampers the model’s ability to make spatially accurate predictions, resulting in higher error rates and reduced accuracy. This further emphasizes the need for the spatial consistency loss in capturing accurate spatial relationships within the trajectory data. As demonstrated in Fig 7, trajectories reconstructed through MTSA-SC exhibit a better alignment with the trajectories of ground truth through adherence to the constraints of the road network. In contrast, predictions lacking spatial consistency loss manifest large trajectory deviations characterized by physiologically implausible shortcuts and destination offsets. This visual comparison demonstrates that spatiotemporal consistency constraints ensure closer adherence to realistic mobility patterns, thereby reducing destination prediction errors and validating the critical role of integrated constraints in joint trajectory completion and prediction.

#### Network hyperparameters experiments.

**Fig 8:**
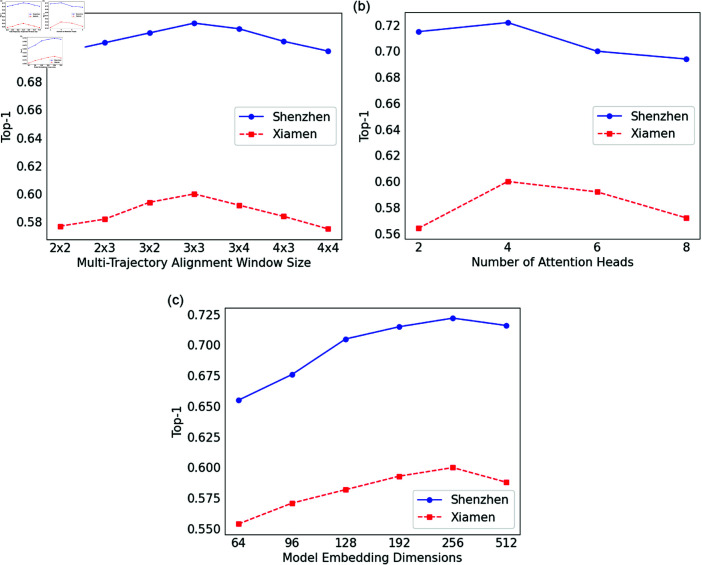
Performance Comparison of Different Hyperparameters.

Fig 8 presents a performance comparison across different hyperparameters, highlighting how each configuration influences the model’s accuracy. It illustrates the impact of key parameters, including multi-trajectory alignment window size, the number of attention heads, and embedding dimensions, on the performance metric Recall@1.

- **Impact of multi-trajectory alignment window size:** As shown in Fig 8(a), the size of the multi-trajectory alignment window determines the local spatial range the model considers when processing trajectory data. To evaluate its effect, we tested various window sizes, including 2×2, 2×3, 3×2, 3×3, 3×4, 4×3, and 4×4. Across both real-world datasets, the Recall@1 metric exhibited an initial increase, followed by a decrease as the window size grew. Smaller windows, such as 2×2, 2×3, and 3×2, restricted the model’s ability to capture local changes and diversity in the trajectories, leading to limited recall performance. As the window size increased to 3×3, the model incorporated more trajectory information, allowing for a better understanding of local patterns and contextual relationships, which led to the highest Recall@1 of 0.72. However, as the window size continued to increase, the computational cost rose significantly, and irrelevant information or noise was introduced, interfering with the model’s learning of key trajectory features, ultimately decreasing recall performance. This suggests that a 3×3 window size strikes the right balance, effectively utilizing local trajectory information while avoiding information overload.
- **Impact of the number of attention heads:** As shown in Fig 8(b), the multi-head attention mechanism captures information from multiple subspaces in parallel, and the number of heads directly influences the model’s ability to represent trajectory features and extract diverse information. We tested the number of heads in the multi-head attention mechanism, setting values of 2, 4, 6, and 8. Across both datasets, Recall@1 reached its peak when the number of heads was set to 4 and gradually decreased with higher values. Fewer heads were unable to fully exploit the rich features and complex relationships within the trajectory data, limiting the model’s performance. However, as the number of heads increased further, the model’s parameter count grew excessively, complicating training and increasing the risk of overfitting. Additionally, the fusion of information across heads became more complex, reducing the model’s ability to effectively utilize the information, leading to decreased recall performance. Hence, for both the Shenzhen and Xiamen datasets, setting the number of attention heads to 4 proved to be optimal.
- **Impact of model embedding dimensions:** As shown in Fig 8(c), the embedding dimension determines how richly the trajectory data is represented in the vector space. To explore its effect, we tested different embedding dimensions: 64, 96, 128, 192, 256, and 512. Across both datasets, Recall@1 steadily increased as the embedding dimension grew, peaking at 256. Therefore, we selected an embedding dimension of 256 as the optimal configuration.

## Conclusion

This paper addresses the challenges of sparse, low-quality trajectory data and spatiotemporal fluctuations in individual travel destination prediction. We propose an improved method that integrates multi-task learning, multi-trajectory subsequence alignment attention mechanisms, and spatial consistency constraint loss functions. Through a multi-task learning framework, we achieve collaborative optimization between trajectory data completion and destination prediction, enhancing model performance under sparse and incomplete data conditions. The introduced multi-trajectory subsequence alignment attention mechanism strengthens the ability to capture local dynamic fluctuations, while the spatial consistency constraint-based loss function improves the spatial rationality of prediction results. Experimental results validate the effectiveness of our proposed method. This approach not only provides an accurate solution for individual travel destination prediction but also holds significant implications for trajectory data mining and intelligent transportation system optimization.

Three strategic directions will propel next-generation trajectory prediction systems: First, developing noise-resilient architectures that synergize meta-learning with causal inference to differentiate sensor anomalies from true mobility patterns. Second, constructing privacy-enhanced frameworks through federated learning with adaptive differential privacy, compliant with the General Data Protection Regulation. Third, engineering lightweight deployment solutions via edge-cloud co-computation for real-time traffic semantics integration. Systematic validation across heterogeneous datasets will strengthen cross-scenario generalization. Complementary investigations will address two fundamental challenges: (1) causal disentanglement of travel behavior determinants versus environmental confounders, and (2) sustainable learning mechanisms adapting to urban evolution without infrastructure overhauls.
